# Hippophagy

**Published:** 1858-03

**Authors:** 


HIPPOPHAGY.—The following characteristic advertisement appeared in the *Courier de Saone et Loire*:—"M. Parent, knacker, informs connoisseurs that from this date to the 8th of May, 1858, he offers horse-flesh, of the best quality only, at the following prices: shoulder, rump-steak, and sirloin, at twenty cents the kilogramme; the chines at reduced prices. It is understood that at this price the weight of bone is included."

